# Circulating and Urinary Concentrations of Malondialdehyde in Aging Humans in Health and Disease: Review and Discussion

**DOI:** 10.3390/biomedicines11102744

**Published:** 2023-10-10

**Authors:** Dimitrios Tsikas, Stefanos A. Tsikas, Marie Mikuteit, Stefan Ückert

**Affiliations:** 1 Core Unit Proteomics, Institute of Toxicology, Hannover Medical School, 30623 Hannover, Germany; 2 Dean’s of Office of Studies, Academic Controlling, Hannover Medical School, 30623 Hannover, Germany; 3 Department of Rheumatology and Immunology, Hannover Medical School, 30623 Hannover, Germany; 4 Dean’s Office, Curriculum Development, Hannover Medical School, 30623 Hannover, Germany; 5 Department of Urology and Urological Oncology, Division of Surgery, Hannover Medical School, 30623 Hannover, Germany

**Keywords:** aging, blood, creatinine, disease, drugs, females, health, males, malondialdehyde (MDA), oxidative stress, urine

## Abstract

(1) Background: Malondialdehyde (MDA) is a major and stable product of oxidative stress. MDA circulates in the blood and is excreted in the urine in its free and conjugated forms, notably with L-lysine and L-serine. MDA is the most frequently measured biomarker of oxidative stress, namely lipid peroxidation. Oxidative stress is generally assumed to be associated with disease and to increase with age. Here, we review and discuss the literature concerning circulating and excretory MDA as a biomarker of lipid peroxidation in aging subjects with regard to health and disease, such as kidney disease, erectile dysfunction, and COVID-19. (2) Methods: Scientific articles, notably those reporting on circulating (plasma, serum) and urinary MDA, which concern health and disease, and which appeared in PubMed were considered; they formed the basis for evaluating the potential increase in oxidative stress, particularly lipid peroxidation, as humans age. (3) Results and Conclusions: The results reported in the literature thus far are contradictory. The articles considered in the present study are not supportive of the general view that oxidative stress increases with aging. Many functions of several organs, including the filtration efficiency of the kidneys, are physiologically reduced in men and women as they age. This effect is likely to result in the apparent “accumulation” of biomarkers of oxidative stress, concomitantly with the “accumulation” of biomarkers of an organ’s function, such as creatinine. How free and conjugated MDA forms are transported in various organs (including the brain) and how they are excreted in the urine via the kidney is not known, and investigating these questions should be the objective of forthcoming studies. The age- and gender-related increase in circulating creatinine might be a useful factor to be taken into consideration when investigating oxidative stress and aging.

## 1. Introduction

**Figure 1:**
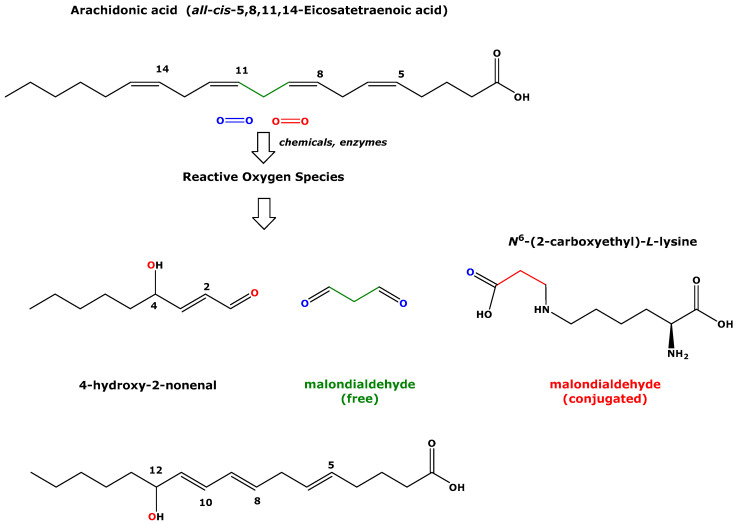
Simplified scheme showing the chemical structures of arachidonic acid, a polyunsaturated fatty acid, which is peroxidized with chemicals and enzymes such as cyclooxygenase, to finally generate 4-hydroxy-2-nonenal and malondialdehyde (MDA) in its free form. MDA also occurs as a conjugate with the terminal amine group (*N*^ε^) of L-lysine residues in proteins, such as albumin and hemoglobin [3]. 12-Hydroxy-5,8,10-heptadecatrienoic acid and thromboxane A_2_ (not shown) are also metabolites of arachidonic acid peroxidation [1].

Malondialdehyde (MDA) is one of the most and best-investigated biomarkers of oxidative stress, notably lipid peroxidation [1] (Table 1). Reactions using chemicals and enzymes, such as the peroxidation of arachidonic acid and other polyunsaturated fatty acids (PUFA) induced by cyclooxygenase (COX), lead to the formation of MDA and other peroxidized species [1] (Figure 1). MDA is a dicarbonylic substance (1,3-propanedial), it exists in its free non-conjugated form, and it is conjugated with low-molecular-mass compounds and high-molecular-mass compounds, including proteins (Figure 1). Free and conjugated MDAs circulate in the blood and are excreted in the urine. Blood (i.e., plasma and serum) is the most frequently analyzed biological sample for MDA (Table 1). The concentration of the sum of free and conjugated MDA species is most frequently determined via the so-called thiobarbituric acid reactive species (TBARS) assay. In combination with a chromatographic method, such as high-performance liquid chromatography (HPLC), the TBARS assay is more specific, and it mainly determines free MDA. The concentration of the free form of MDA in plasma, serum, and urine can be determined using gas chromatography–mass spectrometry (GC-MS) methods, especially after highly specific derivatization with pentafluorobenzyl bromide [2]. This method utilizes the C-H-acidity of the methylene group of MDAs. Based on measurements in biological fluids and tissues, MDA is considered to impact human life and health, and it may be involved in numerous diseases associated with various organs and in conditions such as hypertension and pregnancy (Table 1).

Accurate measurement of MDA, especially in lipid-rich biological samples such as plasma and serum, is challenging because it is associated with many analytical and pre-analytical shortcomings. A major pre-analytical challenge is sampling and sample storage of plasma and serum samples because of the abundant artefactual formation of MDA form “spontaneously” peroxidizing lipids, the main origin of MDA [1,2]. This is of particular relevance in long-term clinical and nutritional studies [2]. Artefactual formation of MDA and other lipid-derived biomarkers of oxidative stress, such as the F_2_-isoprostanes, is almost absent in urine, which is practically free of lipids [1]. MDA, other aldehydes such as 4-hydroxy-2-nonenal (Figure 1), and the F_2_-isoprostanes are biochemically closely associated with each other [1]. Yet, in contrast to MDA, for which only scarce pharmacological and toxicological data are available, the particular F_2_-isoprostane 15(*S*)-8-*iso*-prostaglandin F_2α_ possesses biological and pharmacological properties [4]. Based on the currently available data, MDA seems to be a “pure” biomarker of oxidative stress in humans.

As well as biological complexity and variation, and the use of different analytical methods that have been used in human studies, especially in consideration of the numerous analytical and pre-analytical shortcomings that artefactually increase MDA concentration in plasma and serum upon sample storage, there are major reasons for the reportedly highly divergences in MDA concentrations [1,5]. These reasons are likely to have contributed to the discrepant reported observations regarding the effects of antioxidants such as ascorbic acid, which should be able to decrease MDA concentration upon supplementation [5]. More than 200 theories were formulated to explain the process of aging [6]. The free radical theory of aging proposed by D. Harman in 1956 [7] is the most popular and widely used. In accordance with this theory, oxidative stress and associated damage increase with aging due to an imbalance between the formation and elimination of free radicals, which impairs physiological functions, finally leading to disease [7,8]. Oxidative stress is a very complex and insufficiently understood biological phenomenon. Nevertheless, oxidative stress is thought to be central to human life and to be closely associated with disease and aging. In about eight percent of research articles reporting on oxidative stress and aging, MDA has been investigated as a biomarker of oxidative stress (Table 1).

In this article, we review and discuss scientific work published in the last two decades and archived in the PubMed data bank (https://pubmed.ncbi.nlm.nih.gov (accessed on 15 August 2023)). We focus on issues related to biological variation, i.e., health, disease, and lifestyle, such as sport and nutrition, including supplementation of antioxidants (Table 1). Our special focus is on the effects of aging in male and female humans on circulating and excretory MDA concentrations in health and disease. We considered research work in which the analytical methodology used to measure MDA and TBARS has been satisfactorily reported. To examine potential associations of aging and oxidative stress, we considered large cohort studies on humans in health and disease, notably including kidney diseases that are often accompanied by diabetes and various cardiovascular diseases and COVID-19, by which young and elderly people of both sexes were affected globally. We also considered erectile dysfunction, which affects mostly elderly men. In the sections that follow, we review and critically discuss discrepant published research observations and conclusions. As telomere length is considered a biomarker of biological age, we also treated potential associations between telomere length and aging. Eventually, we included in our work rarely considered issues such as the effects of drugs on MDA formation.

## 2. Malondialdehyde in Young and Elderly Male and Female People

A cohort of 742 people aged between 65 and 95 years included 309 men and 433 women [9]. Plasma (EDTA) samples were frozen and stored at −80 °C analysis. Plasma MDA was measured as TBARS-adduct (MDA-TBA_2_) by HPLC with visible detection (532 nm). The plasma MDA concentrations were reported to be about 2 µM in non-frail subjects, about 2.5 µM in pre-frail participants, and about 3.6 µM in frail persons [9]. These plasma MDA concentrations are within reference intervals that were established by Nielsen et al. using the same HPLC methodology (MDA-TBA_2_) [10]. MDA plasma concentrations increased with frailty status (*p* < 0.001) but did not correlate with the age or sex of the older adults. The absence of differences between men and women was explained by assuming the loss of estrogen-activating effects on antioxidant enzymes more than ten years after menopause [9].

Nielsen et al. found slightly (by 20%) but significantly higher (EDTA) plasma MDA concentrations in men (*n* = 107, 20–79 years) compared to women (*n* = 106, 21–79 years), but no associations with the volunteers’ age were found [10]. Nielsen et al. found that smoking and alcohol consumption were associated with higher plasma MDA levels [10]. In six persons of the same study, plasma MDA concentration (0.39 µM to 0.59 µM) varied between 6% and 30% on six consecutive days. Nielsen et al. reported MDA concentrations in serum and plasma of 13 subjects (serum, 1.16 µM), citrated plasma (0.89 µM), heparinized plasma (1.27 µM), and EDTA plasma (0.26 µM) [10]. These observations contradict MDA concentrations measured by gas chromatography–mass spectrometry (GC-MS) in serum (0.42 µM), heparinized plasma (0.59 µM), and EDTA plasma (2.76 µM) [1].

Miller et al. [11] measured serum MDA concentrations in 123 healthy elderly adults (aged about 50 years) by a batch spectrophotometric TBARS assay [1]. They found higher MDA serum concentrations in smokers compared to non-smokers, yet they did not find a correlation after Spearman between age and MDA serum concentration, which was slightly but significantly higher (by 7%) in men compared to women (21.9 µM versus 20.4 µM, *p* = 0.001) [11]. MDA serum concentrations of the order of 20 µM are considered high [1,10].

MDA plasma concentrations were measured in 123 healthy humans and found to be higher in smokers compared to non-smokers [12]. In the whole group, no correlation was found between MDA concentration and age. However, female smokers had the highest rate of MDA increase with age, followed by male smokers and female non-smokers. MDA levels of male non-smokers were unaffected by age [12].

Campesi and colleagues measured MDA in the serum of 40 aging healthy women (fertile, mean age, 36 years); postmenopausal, mean age, 55 years and 45 men (mean age, 34 years; mean age, 54 years) by means of a batch TBARS assay [13]. Mean MDA serum concentrations were reported to range between about 10 and 22 µM. A significant positive correlation between age and MDA concentration was observed only in men before body weight correction (*r* = 0.369; *p* = 0.0011) [13]. In women, serum IL-6 concentration correlated with age (*r* = 0.426; *p* < 0.001) without body weight correction; *r* = 0.457; *p* < 0.001, with body weight correction. In postmenopausal women, serum MDA concentration was found to predict the serum concentration of asymmetric dimethyl arginine (ADMA) and vice versa both before and after weight correction [13]. ADMA is an endogenous strong inhibitor of neuronal nitric oxide synthase activity [14]. ADMA is produced by post-translational modification. The authors of this study concluded that “aging/menopausal status increased many more cardiovascular risk factors in women than aging in men, confirming that postmenopausal women had increased vascular vulnerability and indicating the need for early cardiovascular prevention in women. Sex-gender differences are also influenced by body weight, indicating as a matter of debate whether body weight should be seen as a true confounder or as part of the causal pathway” [13].

Miquel and colleagues measured the MDA plasma concentration in 50 men and 50 women by means of a batch TBARS assay [15]. Unfortunately, the study cohort has not been sufficiently detailed. In the age group 21–40 years, men were found to have higher (by 27%) MDA concentrations than women (2.46 ± 1.09 µM vs. 1.79 ± 0.66 µM). In the age group 41–60 years, men were found to have slightly higher (by 5%) MDA concentrations than women (2.99 ± 1.62 µM vs. 2.84 ± 1.67 µM). In the older group (61–70 years), men were found to have considerably (by 48%) lower MDA concentrations than women (2.41 ± 1.61 µM vs. 4.54 ± 1.67 µM) [15]. The authors of the study stated that their data show a very strong positive correlation between age and plasma MDA concentration in women.

Huerta and coworkers measured the MDA plasma concentration in 154 non-smoking Spanish elderly without major illness [67 men (mean age, 72 years) and 87 women (mean age 74 years)], who resided in seven nursing homes in Northern Spain, by means of a batch TBARS assay [16]. MDA was measured using a commercially available assay (LPO-586, Byoxytech, Oxis International S.A., Bonneuil sur Marne Cedex, France) after deproteinization with trichloroacetic acid and centrifugation. MDA plasma concentration was measured to be 2.0 ± 1.5 µM. These authors stated that plasma MDA is an independent predictor of mortality. Low plasma MDA concentrations and high plasma α-tocopherol concentrations were found to be associated with the lowest mortality. Previously, in a Finnish elderly population (*n* = 480, age ≥ 65 years), MDA was measured in serum by a batch spectrophotometric TBARS assay. Low MDA concentrations were found to be associated with an increased risk of non-vascular mortality. However, with only age adjustment, this statistical association was not observed [17].

Toto and colleagues performed a meta-analysis study of urinary MDA in the general population [18]. They included 35 studies in which MDA concentrations were determined in human urine by GC or HPLC coupled with mass spectrometry (MS), fluorescence detection, or UV spectrophotometry. Urinary MDA concentrations were corrected for creatinine concentrations, and the data were presented in mg MDA/g creatinine, with a value of 0.637 mg MDA/g creatinine corresponding to a value of 1 µmol MDA/mmol creatinine. The geometric mean of urinary MDA concentration was 0.10 mg/g creatinine with a 95% percentile confidence interval of 0.07–0.12 mg/g. Based on this meta-analysis, creatinine-corrected MDA excretion rate was found to increase with age significantly (*p* = 0.041) but to be independent of sex [18]. A limitation of that meta-analysis is that none of the included individual studies covered the whole age range (<30 years to >50 years). Another limitation of that study is the lack of homogeneity in data collection. Toto et al. concluded that the values determined in their study should be considered preliminary as they are based on moderate- to low-quality studies [18].

In summary, the data reported in the articles discussed above on circulating and excretory MDA concentrations do not allow the drawing of a definite conclusion regarding aging and MDA concentration in healthy humans. The conclusion drawn by Toto et al. [18] in their meta-analysis paper for urinary MDA can be extended to circulating MDA. As discussed by us previously [1,2], both analytical and pre-analytical shortcomings are likely to have contributed to contradicting results and conclusions. As will be discussed below, the general expectation of increasing oxidative stress with increasing age is presumably arbitrary and wishful thinking rather than evidence-based [5].

## 3. Malondialdehyde in Kidney Disease

By means of a batch spectrofluorometric TBARS assay, Li and coworkers measured MDA in EDTA plasma samples of 2169 Chinese men and women (age, 45 ± 14 years; range: 20–80 years for women, 20–90 years for men) [19]. The MDA plasma concentration was reported to be 4.6 ± 1.1 µM. Males (*n* = 1371) had slightly (by 6%) but significantly higher MDA plasma concentrations than females (*n* = 758): 3.63 µM vs 3.41 µM. Young (age < 25 years) and adults (age 25–65 years) had lower MDA plasma concentrations than older humans (age ≥ 65 years). Estimated glomerular filtrate rate (eGFR), a measure of kidney function, correlated inversely with age in males (*r*^2^ = 0.366) and females (*r*^2^ = 0.434) [19]. MDA plasma concentration correlated after Pearson inversely with eGFR without adjustment (*r* = −0.170) and after adjustment for age and gender (*r* = −0.152) and numerous other confounding factors (*r* = −0.142). The study by Li and colleagues clearly showed that kidney function measured as eGFR declines with age of the Chinese males and females and that plasma MDA concentration increases mainly due to impaired kidney function and grade of impairment rather than due to aging [19].

MDA concentration was measured in the plasma of 604 stable renal transplant recipients (RTR) of Caucasian ethnicity by means of a spectrofluorometric TBARS assay after extraction of TBA-MDA_2_ into butanol [20]. The RTR cohort consisted of 331 males and 273 females with a mean ± standard deviation age of 51 ± 12 years (range, 20–80 years). eGFR was 47 ± 16 (mL/min/1.73 cm^2^). Median MDA plasma concentration was determined to be 5.4 µM (interquartile range, 4.3–6.5 µM). In this RTR cohort, MDA plasma concentration correlated after Spearman with the age of the patients (*r* = 0.148, *p* < 0.001; personal communication by M. Yepes-Calderón).

That study revealed that MDA plasma concentration was significantly associated with the risk of cardiovascular mortality, independent of adjustment for potential confounders, including renal function. Interestingly, this association was stronger in RTRs with relatively lower plasma ascorbic acid (vitamin C) concentrations or relatively lower eGFR values [20]. Vitamin C depletion was found to be associated with all-cause mortality in RTR [21]. The plasma concentration of *N*^ε^-(carboxymethyl)lysine (1.8 [1.5–2.1] µM), an advanced glycation end-product (AGE) of post-translational modification of proteinic L-lysine [22], emerged as a strong determinant of MDA plasma concentration.

**Figure 2:**
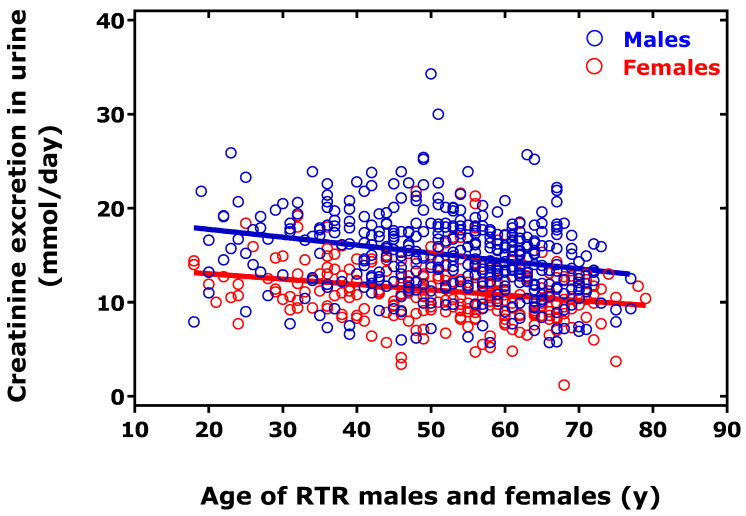
Relationship between excretion rate of creatinine in the urine and age of the male (*n* = 397) and female (*n* = 299) renal transplant recipients (RTR). This Figure was constructed using previously reported data on creatinine [2]. For more details, see the text.

In another study, we measured the urinary excretion rate of MDA in 448 RTR and healthy kidney donors prior to (*n* = 44) and after (*n* = 43) donation of a kidney [2]. In the healthy kidney donors, the median MDA excretion rate was higher (by about 30%) before donation compared to after donation (2.28 µmol/24 h vs. 1.60 µmol/24 h, *p* = 0.005; 0.19 µmol/mmol creatinine vs. 0.13 µmol/mmol creatinine, *p* = 0.005). RTR had higher MDA excretion rates than healthy donors (4.39 µmol/24 h, *p* = 0.046; 0.31 µmol/mmol creatinine, *p* = 0.046). MDA excretion rate (in µmol/24 d) did not depend on the age of the males (*r* = −0.075, *p* = 0.339) and females (*r* = 0.001, *p* = 0.992), as well as in the whole cohort (*r* = 0.029, *p* = 0.543). Creatinine excretion rate differed between males and females RTR (15.1 mmol/d vs. 10.8 mmol/d, *p* < 0.0001) and correlated after Spearman inversely with the age of males (*r* = −0.279, *p* < 0.0001) and females (*r* = −0.251). Linear regression analysis between creatinine excretion (mmol/d, *y*) and age (*x*) resulted in straight lines with regression equation *y* = 19.4 − 84 × *x*, *r* = 0.251 for males and *y* = 14.2 − 57 × *x*, *r* = 0.240 for females (Figure 2). The creatinine-corrected excretion rate of MDA was positively associated with the age of the men (*r* = 0.135, *p* = 0.0234) but not with the age of the women (*r* = 0.060, *p* = 0.445) or with the age of the whole cohort (*r* = 0.071, *p* = 0.133). These results suggest that the MDA excretion rate in the urine may increase with the age of humans due to an age-dependent decrease in kidney function.

In a small cohort of Korean hemodialysis subjects (*n* = 39), the serum concentration of MDA was measured by a commercially available spectrophotometric TBARS assay [23]. In this study, median MDA serum concentration was found to correlate with the coronary artery calcification (CAC) score [6.9 µM in minimal CAC (*n* = 11, 8.2 µM) in mild-to-moderate CAC (*n* = 10), 9.3 µM in severe CAC (*n* = 18), *p* = 0.032]. The serum concentration of MDA positively correlated with the serum concentration of C-reactive protein and, inversely, with the serum concentration of albumin. Age-dependency of MDA serum concentration was not investigated in that study [23].

During physiologic aging, the kidney experiences a progressive functional decline, which leads to an increased risk of acute and chronic kidney disease. There is no convincing evidence that oxidative stress is a major contributor to chronic kidney disease. A Cochrane review did not find a clear benefit of antioxidant supplementation therapies in reducing cardiovascular mortality in patients with chronic kidney disease [24]. Other factors, such as inflammation of the kidney tubular system, have been proposed to play an important role in the underlying aging process [25].

## 4. Malondialdehyde in COVID-19

### 4.1. Malondialdehyde in Acute, Long, Ex and Hospitalized COVID-19 Humans

Circulating and excretory MDA concentrations have been determined in COVID-19 patients and used as a biomarker of oxidative stress [26,27,28,29,30,31,32,33,34]. Of these articles, only one study reported on age-dependency of circulating MDA [34].

In COVID-19 patients older than 65 years, MDA was measured in plasma by a batch spectrophotometric TBARS assay. In 61 patients with a mean age of 83 years, the mean plasma concentration of MDA was determined to be 2.9 µM. MDA differences were found in the comparison groups in fatal and non-fatal events during hospital admission. Also, MDA concentrations were reported to correlate with fatal events after discharge [26].

In 165 hospitalized COVID-19 patients older than 18 years, MDA was measured in plasma spectrophotometrically. Non-survivors (56 subjects) were reported to have higher plasma MDA median concentrations than survivors (0.50 µM vs. 0.36 µM, *p* = 0.01) [27]. The authors of that study concluded that MDA is an independent indicator of a worse prognosis in COVID-19 patients requiring hospitalization.

A prospective cohort study was performed on 98 COVID-19 patients with mild (*n* = 28; median age, 43 years), moderate (*n* = 23; 58 years), and severe (*n* = 39; 61 years) pneumonia [28]. The median MDA plasma concentration was about 0.41 µM, 0.50 µM, and 1.26 µM, respectively; *p* < 0.0001). Deceased patients (*n* = 18) were reported to have higher median MDA plasma concentrations than recovered patients (*n* = 80) (1.24 µM vs. 0.51 µM, *p* < 0.001). The authors of that study concluded that elevated lipid peroxidation might contribute to the severity of COVID-19 [28].

MDA was measured by GC-MS using d_6_-benzaldehyde as an internal standard in the plasma of 88 COVID-19 patients and 33 healthy humans [29]. Healthy subjects (24 females, 9 males; age 45 ± 13 years) served as controls. Of the COVID-19 patients, 66 (25 females, 41 males; age 59 ± 9 years) recovered, and 22 (13 females, 9 males, age 72 ± 7 years) died. Mean MDA plasma concentrations were measured to be about 1.8 µM in the healthy subjects, 2.8 µM in the recovered, and 3.6 µM in the deceased COVID-19 patients [29].

Šķesters and colleagues [30,31] measured MDA in the plasma of 120 COVID-19 patients (age not reported) by means of a commercially available OxiSelect™ TBARS (MDA Quantitation) assay kit. The MDA plasma concentration was reported to be 27 ± 11 µM in 40 acutely ill COVID-19 patients, 31 ± 19 µM in 40 post-COVID-19 patients in the spring-summer wave, and 27 ± 11 µM in 40 post-COVID-19 patients in the summer-autumn wave (no year reported). These are extremely high MDA concentrations [1,10].

Peleman and coworkers measured MDA by a colorimetric assay in the plasma of 120 COVID-19 patients (age range, 24–81 years), in 39 healthy subjects (age range, 25–64 years), and 20 post-operative controls (age range 31–66 years) [32]. MDA plasma concentrations were higher in the COVID-19 patients (range, 1–3 µM log_2_-transformed) compared to the healthy subject range, 1–2 µM log_2_-transformed (*p* < 0.001). The authors of that study stated that the rise in MDA levels in COVID-19 during intensive care unit (ICU) admission could be attributed to recurrent ischemia and ischemia-reperfusion damage, loss of control over the unbound iron pool, micro-emboli and a pro-oxidative burst of neutrophils [32]. The authors of that study also stated that MDA is a marker for ferroptosis. The term ferroptosis was coined in 2012 to describe an iron-dependent regulated form of cell death caused by the accumulation of lipid-based reactive oxygen species [32].

**Figure 3:**
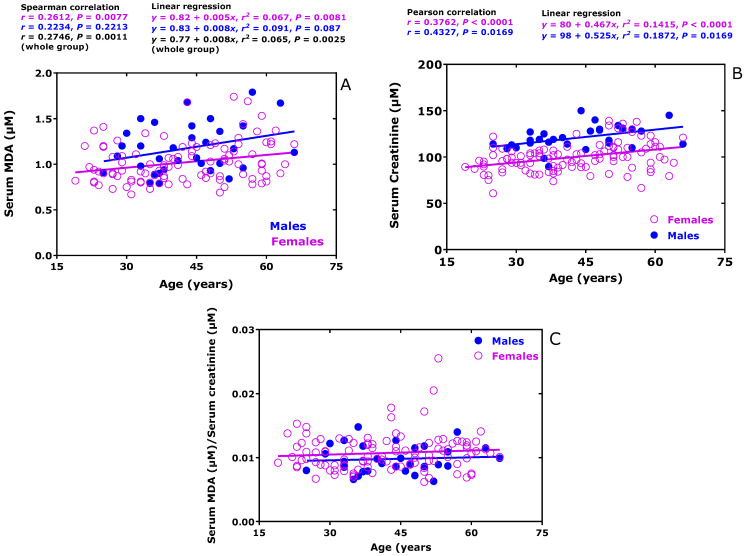
Relationship between serum MDA concentration (**A**), serum creatinine concentration (**B**), or the molar serum MDA-to-serum creatinine ratio (**C**) with the age of the COVID-19 volunteers of the study. These Figures were prepared using previously reported data on MDA and creatinine [34]. In (**C**), there was no correlation and no linearity.

In human subjects with ex-COVID (*n* = 24) and long-COVID (*n* = 124), we measured by GC-MS very similar serum MDA concentrations of 1.11 ± 0.22 µM and 1.10 ± 0.38 µM in these groups [34]. The concentration of MDA in serum correlated after Spearman with the age of the women of study (*r* = 0.2612, *p* = 0.0077) but not with that of the men (*r* = 0.2223, *p* = 0.2214), while consideration of the whole cohort revealed correlation (*r* = 0.2746, *p* = 0.0011) (Figure 3A). Linear regression analysis between MDA serum concentration (*y*) and age (*x*) resulted in straight lines with very small slope values of 0.005 µM MDA/year for females and 0.008 µM MDA/year for males (Figure 3A). In the same cohort, we found correlations after Pearson between serum creatinine and subject age in the female (*r* = 0.3762, *p* < 0.0001) and in the male (*r* = 0.4327, *p* = 0.0169) groups (Figure 3B). These observations suggest that the age-dependency of circulating MDA may result from a decreased excretion of free MDA in the urine, analogous to circulating creatinine. The serum MDA/creatinine molar ratio values did not differ between males (0.0098 ± 0.0004, *n* = 30) and females (0.01069 ± 0.00026, *n* = 115). There was neither correlation nor linearity between circulating MDA/creatinine ratio and age (Figure 3C). Thus, serum creatinine could serve as a suitable corrector for circulating MDA and perhaps for other metabolites that are excreted in the urine (see also below).

### 4.2. Potential Effects of Age, Gender, and Serum Creatinine Concentrations on MDA Serum Concentration in COVID-19

To test for potential effects of age, gender, and serum creatinine concentrations on the MDA serum concentration of the patients of the COVID-19 study, we performed ordinary least squares (OLS) linear, multivariate regression analysis. The results of these analyses are summarized in Table 2 and illustrated in Figure 3.

**Figure 4:**
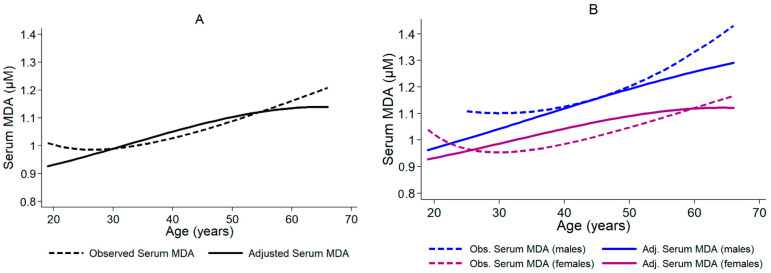
Polynomial fits between serum MDA concentration and age of the subjects in the COVID-19 study. (**A**) Full sample with 133 observations. The dashed line is the polynomial fit for the subject age and the observed serum MDA concentrations. The solid line shows the polynomial fit for age and predicted serum MDA values based on the regression Model (2) in Table 2 and adjusted for serum creatinine, gender, and age. (**B**) Split sample for females (*n* = 102, purple curves) and males (*n* = 31, blue curves). Dashed curves are polynomial fits for age and observed serum MDA concentration; solid curves are polynomial fits for age and predicted serum MDA concentration. The predicted values are based on separate linear regression analyses (by gender) with age and serum creatinine concentration as explanatory variables. STATA 14 (StataCorp, College Station, TX, USA) was used for multiple linear regression analyses (Table 2, Figure 4).

The solid curve in Figure 4A is a polynomial fit of predicted MDA serum concentrations and subjects’ age. The dashed line in Figure 4A is a polynomial fit for observed MDA serum concentration and subjects’ age. The solid and dashed curves in Figure 4A show a positive correlation between MDA serum concentration and the age of the subjects. However, adjusting for creatinine serum concentration particularly alters the left and right tail of the solid curve because creatinine levels are high in older subjects and low in young persons. Since confidence intervals are quite wide (not shown in Figure 4), the curves do not significantly differ from each other at any given age.

Model (1) of Table 2 reveals a statistically significant yet very weak positive association between subjects’ age and MDA serum concentration: one additional year of life correlates with an increase of serum MDA concentration by 0.005 µM. Model (1) also indicates that females in the sample have significantly lower MDA serum concentrations than males. As serum creatinine concentration correlates with both MDA serum concentration (*r* = 0.424, *p* < 0.001) and age (*r* = 0.258, *p* = 0.003), the effect of serum creatinine concentration on the age-dependence of the MDA serum concentration was investigated with Model (2). An increase in serum creatinine concentration by 1.0 µM is significantly associated with a rise of 0.005 µM in serum MDA concentration. When controlling for serum creatinine concentration, the effect of an additional year of life on the increase in MDA serum concentration diminishes to 0.003 µM, and differences in MDA serum concentrations between males and females are no longer statistically significant. Therefore, according to Model (2), creatinine serum concentration not only exhibits a correlation with MDA serum concentration but likely also acts as a moderator for the influence of age and gender on the dependent variable.

In Figure 4B, we ran separate regressions for males and females and plotted the predicted serum MDA concentrations against the respective observed values, analogous to Figure 4A. The solid curves (adjusted for serum creatinine concentration) suggest that the increasing difference in MDA serum concentrations between females and males can partly be explained by the age-dependent increase of creatinine serum concentration, which concerns men to a slightly higher degree than women.

## 5. Malondialdehyde and Erectile Dysfunction

In Westernized countries, erectile dysfunction (ED) is the most common sexual disorder among the male population. According to data from community-dwelled epidemiological surveys and projections, by 2025, more than 300 million men over the age of 18 of all ethnic societies are predicted to experience several degrees of ED. ED is defined as the persistent inability to achieve or maintain an erection adequate for sexual intercourse (vaginal penetration) with the female partner. The prevalence is about 40% in men in their fourth decade of life and 67% in those in their seventh decade. The disorder is commonly associated with various physiological comorbidities, including cardiovascular diseases, diabetes mellitus, or traumatic lesions to pelvic nerves as a result of prostatectomy or cystectomy surgery [35,36].

Since the early 1980s, basic research studies on the physiology of penile erection and the pathophysiology of ED have significantly broadened our knowledge. Aside from the classical transmitters of the sympathetic and parasympathetic nervous system, non-adrenergic and non-cholinergic (NANC) factors are also involved in the control of the function of penile erectile tissue (vascular and non-vascular corpus cavernous smooth muscle). Studies have implicated the role of hormones (such as testosterone, dihydrotestosterone, cortisol, human growth hormone), as well as various endogenous bioactive peptides (for example, vasoactive intestinal polypeptide, angiotensin, bradykinin, calcitonin gene-related peptide, endothelin 1 and oxytocin) in mediating the male sexual response cycle including penile erection [37,38]. In particular, the discovery of the gaseous neurotransmitter nitric oxide (NO) and the signaling molecule cyclic guanosine monophosphate (cyclic GMP) as the major effectors in penile smooth muscle relaxation, which is the crucial step in achieving an erection, has led to the identification of certain drugs administered orally or by intracavernous injection. Among these compounds are NO donor drugs, such as linsidomine (SIN-1), and inhibitors of the enzyme degrading cyclic GMP, the phosphodiesterase type 5 (PDE5), such as avanafil, sildenafil, tadalafil and vardenafil [39,40].

With aging, increasing oxidative damage, brought about by the enhanced production of so-called reactive oxygen species (ROS) as a result of, for example, the excess activation of nicotinamide adenine dinucleotide phosphate (NADPH) oxidase, may contribute significantly to the pathophysiology of ED by detrimentally affecting key proteins. These proteins are involved in the NO/cyclic GMP pathway, such as the endothelial and neuronal nitric oxide synthases (eNOS/nNOS), the soluble guanylyl cyclases (enzymes known to produce the second messenger cyclic GMP), as well as cyclic nucleotide-dependent protein kinases, involved in the control of membrane permeability. This may lead to an impairment of the activity of said enzymes/receptors, reduced levels of NO, local endothelial and vascular dysfunction, and the subsequent apoptosis of cavernosal smooth muscle cells. The reaction between ROS (e.g., superoxide) and NO forms peroxynitrite, a highly pro-oxidative species capable of further reducing the amount of NO required for penile erection [41,42,43]. Since the peroxidation processes mediated via ROS and transition metals, in particular Fe^2+^, do not only affect cellular proteins but also other vital molecules such as PUFA, an increased production of MDA is likely to occur. Circulating MDA concentrations and tissue MDA contents have been determined in subjects with ED who suffered from other diseases, including diabetes [44,45,46,47,48,49]. Yet, no age-dependency of MDA was investigated explicitly. In addition, the number of human subjects involved in the studies is generally very low. ED is a common complication in patients with diabetes mellitus. In this context, circulating MDA and the NO metabolites nitrite and nitrate are frequently measured as biomarkers of oxidative stress [44].

In a study by Tuncayngin and colleagues, human penile tissue from 22 patients who had undergone penile prostheses implantation surgery was investigated [45]. Eight of the patients were suffering from diabetic ED, and 14 had non-diabetic ED. The mean age was 55 (43–67) years for the diabetic and 59 (38–71) years for the non-diabetic ED group. By means of a spectrophotometric TBARS assay, the MDA content in corpus cavernosum was measured to be 77 ± 2 nmol/g tissue in the diabetic group (*n* = 8) and 55 ± 6 nmol/g tissue in the non-diabetic group (*n* = 14) (*p* < 0.001).

El-Latif and coworkers investigated 26 diabetic patients (range, 33–60 years), 15 impotent patients with psychogenic etiology (range, 32–50 years), and 10 normal potent men of the same age range [46]. In plasma, mean concentrations of MDA in venous blood were measured to be 3 µM in the diabetic group, 1 µM in the psychogenic group, and 1 µM in the healthy controls. Similar MDA levels were measured in plasma from cavernosum blood. It turned out that MDA can presumably serve as a predictor of low peak systolic velocity (PSV) and the diminished percentage increase in the diameter of the cavernosal central artery (CAD), thus correlating with the severity of ED (negative correlation between levels of MDA in the systemic and penile blood and PSV as well CAD increase).

Hamdan and Al-Matubsi investigated 56 diabetic patients with ED (age range, 17 to 58 years) and measured MDA plasma concentrations [47]. Mean MDA plasma concentration was 0.46 µM in the control group (*n* = 30; age range, 19–55 years) and 1.27 µM in the diabetic ED patients (*p* < 0.001).

Serefoglu and colleagues measured MDA in the plasma of 58 patients (age range, <10 years (*n* = 8) to 60 years (*n* = 10) with ED by a batch TBARS assay [48]. In penile blood plasma, the mean MDA concentration was 12.8 µM and correlated positively with the mean peak systolic velocity (*r* = 0.159) and the end-diastolic velocity (*r* = 0.151) and inversely with the resistive index (*r* = −0.116) of cavernosal arteries [48].

In a study on patients with ED treated with sildenafil, a phosphodiesterase type 5 (PDE-5) inhibitor, MDA was measured in plasma by a batch TBARS assay in 26 ED patients (age, 56 ± 13 years), 18 patients with ED and diabetes (age, 57 ± 9 years), and in 28 healthy subjects (age, 54 ± 6 years) [49]. The corresponding MDA plasma concentrations were reported as 6.0 ± 2.1 µM, 6.6 ± 2.1 µM, and 6.2 ± 1.9 µM with no differences between the groups.

Given the few data currently available, further research efforts are indicated to add convincing evidence to the hypothesis that MDA might be a reliable biochemical diagnostic marker for the evaluation of penile arterial insufficiency due to diabetic and, consequently, vasculogenic/neurogenic ED. Although some studies have proposed a relationship between ED and oxidative stress, it remains to be proven whether or not a deficiency in the production of NO, a lack of defensive antioxidative mechanisms, and an increase in circulating MDA (possibly due to the peroxidation of membrane lipids) may contribute to the development of ED [44,50]. Whether ED is associated with an age-dependent increase in MDA remains to be investigated. Furthermore, the reducing capacity of antioxidants, either of synthetic origin or naturally occurring (flavonoids, terpenoids, carotenoids, alkaloids, and polyunsaturated fatty acids), to ameliorate via scavenging free radicals and facilitate the chelation of transition metals (possibly via electron donation) oxidative stress and prevent the production of oxidized intermediates of lipid peroxidation requires further investigations [51]. In the future, the inhibition or modulation of lipid peroxidation by various bioactive compounds may emerge as a straightforward approach to effectively treat ED of various origins in a significant number of patients.

## 6. Malondialdehyde and Telomere Length

Telomere length (TL) is considered to be a biomarker of biological age [52]. Yet, the number of studies investigating the in vivo connection between oxidative stress and telomere dynamics is limited (reviewed in [53]).

Demissie and colleagues investigated the relationship between the urinary excretion of 8-*iso*-prostaglandin F_2α_, a biomarker of oxidative stress [1], and the telomere length of leukocytes in 171 human subjects (age range, 40–89 years) of the Offspring cohort of the Framingham Heart Study [54]. MDA was not measured in that study. The terminal restriction fragment (TRF) length was reported to be inversely associated with human age (*r* = −0.41, *p* < 0.0001). Also, age-adjusted TRF length was found to be inversely (*r* = −0.16, *p* = 0.005) correlated with 8-*iso*-prostaglandin F_2α_ (8-*iso*-PGF_2α_, also known as 8-*epi*-PGF_2α_), suggesting that oxidative stress accounts for shorter leukocyte telomere length. This observation confirmed previous findings [55].

Yadav and Maurya investigated correlations between TL and MDA in humans [56]. TL, MDA (by a batch TBARS assay), and glutathione (GSH, an endogenous antioxidant, and cofactor) were measured in leukocytes and erythrocytes, respectively, of 105 healthy subjects of both sexes aged 20–77 years. Women of childbearing age were excluded if they were pregnant or in the postpartum period. TL was found to decrease with age (*r* = −0.405, *p* < 0.0001) [56]. The ratio of TL to MDA was reported to correlate inversely with human age (*r* = −0.719, *p* < 0.001), whereas the ratio of TL to GSH was found to correlate positively with human age (*r* = 0.821, *p* < 0.001). However, the TL/MDA ratio varied strongly in the range of 20–35 years, while almost no decrease in the TL/MDA ratio was observed in the range of 35–77 years [56]. The data of that study does not allow the drawing of a reasonable conclusion regarding the dependency of erythrocytic MDA and the age of the investigated human subjects.

A review and meta-analysis study concluded that it is of utmost importance to conduct further robust studies in humans on the relationships between MDA and TL [53].

## 7. Adducted Malondialdehyde

The metabolism of endogenous and exogenous non-conjugated MDA has been investigated in vitro and in vivo [57,58,59]. Major MDA metabolites have been identified as CO_2_ and acetic acid [57]. Unchanged MDA is also excreted in the urine [57]. Several MDA derivatives have been identified in the urine of animals and humans [60]. Identified MDA metabolites include *N*^α^-acetyl-*N*^ε^(2-propenal)lysine [61], *N*-(2-propenal)serine [62], *N*^ε^-(2-propenal)lysine, which was identified as a major MDA metabolite [63], and *N*-(2-propenal)ethanolamine [64]. Additional metabolites of MDA in human urine include a guanine-malondialdehyde adduct [65] and a deoxyguanosine-malondialdehyde adduct [66].

A review of MDA metabolism and the advanced lipid peroxidation end-product MDA-lysine conjugate in aging and longevity has been recently reported [67]. Jové and colleagues concluded that “The findings in different experimental paradigms such as: (i) Increase in the concentration of MDA-Lys with age within an animal species, (ii) the presence of a lower concentration of MDA-Lys the greater the longevity of the animal species, (iii) its accumulation above physiological levels in a wide variety of pathological conditions, and (iv) the downward modulation of its concentration by nutritional and pharmacological interventions that have shown an extension of longevity, make the MDA-Lys adduct a potential biomarker of aging and longevity” [67]. However, these conclusions are based almost exclusively on data reported on the content of MDA-Lys adduct in tissues in many experimental studies on animals and on only two studies in humans (adults vs. old subjects), in which, moreover, the MDA-Lys adduct was detected by immunohistochemistry and Western blot. No studies have been considered in the review by Jové and colleagues, in which the MDA-Lys was measured in human plasma, serum, or urine [67].

Girón-Calle and colleagues observed that both gastric intubation and intraperitoneal injection of synthetic MDA-Lys to rats resulted in excretion of unchanged MDA-Lys in considerable amounts in the urine (about 31% and 34% of the administered dose, respectively) [68]. To the best of our knowledge, no quantitative data are available for the MDA-Lys adduct in human urine.

## 8. Rarely Considered Issues of Malondialdehyde—Potential (Dual) Effects of Drugs

Acetylsalicylic acid (aspirin) is an irreversible inhibitor of cyclooxygenase (COX), which can generate considerable amounts of MDA of thromboxane A_2_ (TxA_2_) in human platelets [69]. In studies involving aspirin administration, circulating MDA concentration is expected to be decreased, especially at higher aspirin doses (e.g., 500 mg).

Some sulfonamides (RSO_2_NH_2_) that inhibit carbonic anhydrase (CA) activity have been reported to inhibit COX activity as well, most likely because of their sulfonamide group [70]. In vivo in humans, cerebrovascular reactivity of infused acetazolamide was found to correlate inversely with the plasma concentrations of thromboxane B_2_ (TxB_2_) and 11-dehydro-TxB_2_ (stable metabolites of TxA_2_), suggesting inhibition of TxA_2_ formation in the platelets [71]. Whether acetazolamide influences the homeostasis of MDA has not been investigated thus far.

Intravenous injection of acetazolamide, a strong inhibitor of CA (5 mg/kg) in a healthy infant (female, 3.3 years old), increased the excretion of many amino acids [72]. In contrast, intravenous injection of acetazolamide (5 mg/kg) in an infant with cystinuria (female, 1.5 years old) decreased the excretion of many amino acids [62]. These effects disappeared when acetazolamide was repeatedly administered. In the dog, urinary excretion of glutamine, glutamate, and aspartate remained minimal before and after infusion of acetazolamide (priming dose, 20 mg/kg; maintenance dose, 20 mg/kg/h) [73]. These studies indicate that acetazolamide may exert different effects on the reabsorption from the urine of amino acids in health and disease, especially in diseases associated with impaired amino acid metabolism, such as cystinuria [72] and acidosis [73].

In healthy humans, acetazolamide at a therapeutic dose inhibits the reabsorption of the anion nitrite, suggesting that CA is involved in the excretion of nitrite, an inorganic anion [74]. In healthy humans, nitrite and MDA were found to correlate positively in urine [75]. In a pilot study, we investigated the excretion of nitrite and MDA in a healthy female volunteer who was administered a 250-mg acetazolamide-containing tablet. The highest excretion rate of nitrite in the urine was observed 1 h after drug administration, while the excretion rate of MDA decreased continuously until the end of the study. These results suggest that acetazolamide may inhibit the excretion of MDA in the urine, while nitrite excretion is maximal after 1 h, in confirmation of a previous study [74].

MDA and uric acid are organic acids. MDA is a weak acid (C-H-acidic; p*K*_a_, 4.46) [1], i.e., MDA is about 10 times stronger than uric acid (an O-H-acidic acid; p*K*_a_, 5.4). It is well established that uric acid is excreted in the urine by organic anion transporters (OAT) in proximal tubule cells. Whether non-conjugated MDA is excreted in the urine by the mediation of OAT is unknown. In the case that MDA is excreted in the urine by OAT, drugs that inhibit OAT in the kidney would increase the renal excretion of MDA and consequently increase its concentration in the blood.

Febuxostat is considered a selective inhibitor of xanthine oxidoreductase (XOR), an enzyme that catalyzes the formation of uric acid. In Dahl salt-sensitive rats, orally administered febuxostat decreased the urinary excretion of TBARS, which was measured by means of a commercially available colorimetric batch TBARS assay [76]. XOR is also considered to generate superoxide anions, which in turn can mediate lipid peroxidation and increase MDA formation. No data were reported on TBARS in the blood in that study.

A meta-analysis study of 12 clinico–pharmacological trials revealed that febuxostat decreased serum uric acid concentration in patients with chronic kidney disease and hyperuricemia in elderly subjects (mean age, 52–81 years) [77]. This study also revealed that serum creatinine values decreased and eGFR values increased [77]. The studies considered in the meta-analysis did not report on the urinary excretion of uric acid and creatinine, nor on MDA and other TBARS in serum and urine.

Type 2 diabetes mellitus is associated with cardiac and endothelial dysfunction caused by various factors such as insulin resistance, microvascular disease, and elevated oxidative stress. Lambardiari and colleagues investigated the effects of a six-month treatment with liraglutide, a glucagon-like peptide-1 analog, or metformin on arterial stiffness and oxidative stress in 60 subjects with newly diagnosed type 2 diabetes (age 51 years; 70% males) [78]. MDA was measured (matrix not reported) spectrophotometrically with a commercial kit (Oxford Biomedical Research, Rochester Hills, MI, USA). Liraglutide administration resulted in a strong decrease in median MDA concentration (0.92 nM to 0.68 nM, *p* = 0.003), while metformin treatment increased median MDA concentration (0.78 nM to 0.86 nM, *p* = 0.09). Liraglutide but not metformin decreased pulse wave velocity, whereas liraglutide (8.9% to 13.2%, *p* = 0.003) and metformin (8.8% to 11.8%, *p* = 0.033) increased flow-mediated dilation. The difference in MDA was found to be associated with the difference in pulse wave velocity. The authors of that study stated that a lower oxidative stress level resulted in a greater improvement in vascular function [78].

The same research group investigated the effects of clopidogrel and aspirin in 121 (93 males, 28 females, mean age 67.2 years) type 2 diabetes patients with revascularized coronary artery disease [79]. The active form inhibits specifically and irreversibly the P2Y12 subtype of ADP receptor of platelets. A daily dose of 100 mg acetylsalicylic acid per os and a daily dose of 75 mg clopidogrel per os were prescribed to the patients for at least one month before inclusion in the study. MDA plasma concentration was measured spectrophotometrically with a commercial kit (Oxford Biomedical Research, Rochester Hills, MI). The baseline MDA plasma concentration was reported to be 5.25 ± 2.26 (presumably nM) (range, 0.40–11.7), indicating a high inter-variation of about 50% [79]. Patients with high-on-treatment platelet reactivity were found to have a higher incidence of β amyloid levels. MDA levels showed a borderline association with high-on-treatment platelet reactivity (*p* = 0.073) in multivariate analysis.

In the studies discussed above [78,79], no age- or gender-related associations were reported. The MDA concentrations reported in those studies are very low and differ greatly (0.92 nM [78] vs. 5.25 nM [79]) despite the co-administration of aspirin and clopidogrel, which are expected to inhibit (platelet-derived) MDA and have been reported in rabbits [80], rats [81], and in humans [82]. It is worth mentioning that clopidogrel administration suppressed in rats the lithium-induced increase in urinary 8-isoprostane [83], which is also considered a biomarker of oxidative stress closely related to MDA [1].

## 9. Supplementation of the Antioxidant *N*-Acetyl-L-cysteine

An increase in oxidative stress with aging is generally expected. This expectation has previously been thoroughly discussed in a joint effort by several researchers studying oxidative stress and health-related outcomes, as well as the underlying biochemical mechanisms [84]. Despite advantages in the area of oxidative stress research, it is still not known why the antioxidants used so far in clinical trials/therapies did not exert the expected effects [5,85]. There is no convincing evidence for the health benefit of antioxidant supplementation therapies such as *N*-acetyl-L-cysteine (NAC), the precursor of L-cysteine and GSH, in various diseases [5], including chronic kidney disease [23] and rheumatoid arthritis [86]. Reported studies do not allow any valid conclusion about the utility of NAC in rheumatoid arthritis, and administration of NAC to patients with rheumatoid arthritis is not recommended in current European and American guidelines [86]. It is worth mentioning that GSH can even promote cyclooxygenase-induced MDA formation in vitro [87].

Lizzo and colleagues investigated the effects of a supplementation of glycine + NAC, two precursors of GSH, at three different daily doses for 2 weeks (low dose: 2.4 g, medium dose: 4.8 g, or high dose: 7.2 g/day, 1:1 ratio) on oxidative stress in a randomized, controlled clinical trial in 114 healthy volunteers [88]. There was one group of young subjects (*n* = 20, 9 females/11 males; mean age, 31.7 years) and one group of old subjects (*n* = 117, 64 females/53 males; mean age, 65.5 years). MDA was measured in plasma by HPLC with fluorescence detection. Mean baseline MDA plasma concentrations were reported to be 0.136 µM in the young and 0.158 µM in the older subjects (*p* < 0.0001) [88]. MDA plasma concentrations of this order of magnitude (range, 0.236–0.415 µM) were measured by GC-MS in heparinized plasma samples of exercising healthy young athletes supplemented with sodium nitrate [89]. In that study, MDA plasma concentrations were found not to change during exercise. MDA plasma concentrations in the young and old subjects differed by 22 nM (by 14%), which corresponds to an increase of about 0.7 nM per life year [88]. The inter-individual variation of MDA plasma concentration is calculated to be 13% in the young and 12% in the old subjects of the study [88], i.e., of the same order of magnitude of the difference in MDA plasma concentration between young and old subjects. In that study, supplementation of glycine + NAC did not result in changes in MDA plasma concentrations compared to placebo [89]. The authors of that study did not report separate data for the females and males for MDA and other biochemical parameters, including creatinine. The glomerular filtration rate is calculated to be about 16% lower in the group of the old subjects compared to the group of the young subjects [90].

## 10. Conclusions

Oxidative stress is generally expected to increase with aging. This expectation has been thoroughly discussed in a joint effort from several researchers studying oxidative stress and health-related outcomes, as well as the underlying biochemical mechanisms. Despite advantages in oxidative stress research, it is still not known why the antioxidants used so far in clinical trials/therapies have not returned the expected effects. The results of the present study, which are based on reported circulating and excretory concentrations of MDA, a generally accepted and widely used lipid peroxidation biomarker, are not conclusively supportive of the general view that oxidative stress increases with age. Analytical and pre-analytical shortcomings are likely to have contributed to contradicting results and conclusions. Many functions of several organs of the human body, notably including the filtration efficiency of the kidneys, decrease physiologically in men and women with aging. This effect is likely to result in the apparent “accumulation” of biomarkers of oxidative stress concomitantly with the “accumulation” of biomarkers of an organ’s function, such as creatinine, which are eliminated via the kidneys. How free and conjugated MDA forms are transported in various organs, including the kidney and brain, is not known and should be the objective of forthcoming studies. Consideration of the age- and gender-dependent increase in circulating creatinine might be a useful factor to be taken into account when investigating oxidative stress and aging using circulating MDA as a biomarker.

## Figures and Tables

**Table 1:** Summary of a selection of articles related to malondialdehyde published from 1947 to the present (2023), which are archived in *PubMed* (accessed on 15 August 2023).

Search Term	No. of Articles	Search Term	No. of Articles
Malondialdehyde	71,143	Malondialdehyde fatigue	319
MDA	80,647	Malondialdehyde erectile dysfunction	105
TBARS	19,557	Malondialdehyde COVID-19	84
TBARS MDA	1045	Malondialdehyde serum	16,845
Malondialdehyde oxidative stress	38,780	Malondialdehyde plasma	11,108
Malondialdehyde peroxidation	22,513	Malondialdehyde urine	1812
Malondialdehyde biomarker	8606	Malondialdehyde saliva	239
Malondialdehyde aging	2533	Malondialdehyde human serum	5413
Oxidative stress	312,577	Malondialdehyde human plasma	5338
Oxidative stress aging	25,793	Malondialdehyde human urine	855
Malondialdehyde liver	14,271	Malondialdehyde human saliva	157
Malondialdehyde brain	8410	Malondialdehyde humans	913
Malondialdehyde cardiovascular	7761	Malondialdehyde human serum	184
Malondialdehyde kidney	7149	Malondialdehyde human plasma	230
Malondialdehyde diabetes	5369	Malondialdehyde human urine	23
Malondialdehyde cancer	4296	Malondialdehyde human sex	836
Malondialdehyde lung	3596	Malondialdehyde human gender	1103
Malondialdehyde hypertension	1582	Malondialdehyde antioxidants	48,227
Malondialdehyde transplantation	1547	Malondialdehyde drugs	9114
Malondialdehyde pregnancy	1522	Malondialdehyde nutrition	6105
Malondialdehyde stroke	1023	Malondialdehyde supplementation	5513
Malondialdehyde Alzheimer’s disease	1001	Malondialdehyde cyclooxygenase	1263
Malondialdehyde Parkinson	556	Malondialdehyde sport	1189
Malondialdehyde rheumatism	397	Malondialdehyde ferroptosis	555

**Table 2:** Ordinary least squares linear, multivariate regression analysis of serum MDA concentration with age, gender, and serum creatinine concentrations in the patients of the COVID-19 study [33].

Model	Dependent Variable	Age (Years)	Gender	Creatinine (µM)	N	R^2^
1	MDA (µM)	0.005 ** (0.002) ^a^	−0.146 ** (0.048) ^a^		133	0.135
2	MDA (µM)	0.003 * (0.001) ^a^	−0.047 (0.063) ^a^	0.005 ** (0.002) ^a^	133	0.196

^a^ Robust standard errors are given in parentheses. “Gender” is a binary indicator that takes the value = 1 for females and the value = 0 for males. *, *p* < 0.05; **, *p* < 0.01.

## Data Availability

Not applicable.
